# Supplementary material for: Antibacterial Activity of Manganese Dioxide Nanosheets by ROS-Mediated Pathways and Destroying Membrane Integrity
Source: Nanomaterials (Basel). 2020 Aug 7;10(8):1545. doi: 10.3390/nano10081545 (PMC7466589; doi:10.3390/nano10081545)
Supplement: Supplementary file 1 [file nanomaterials-10-01545-s001.pdf]

Supplementary Materials

Antibacterial Activity of Manganese Dioxide Nanosheets by ROS-mediated Pathways and Destroying Membrane Integrity

Ting Du [1], Siya Chen [1], Jinyu Zhang [1], Tingting Li [2], Ping Li [1], Jifeng Liu [1], Xinjun Du [1,*] and Shuo Wang [1,3,*]

[1] State Key Laboratory of Food Nutrition and Safety, College of Food Science and Engineering, Tianjin University of Science and Technology, Tianjin 300457, China; tingdu@tust.edu.cn (T.D.); siycmail@163.com (S.C.); jinyuzhang@mail.tust.edu.cn (J.Z.); zoelxx@tust.edu.cn (P.L.); liujifeng111@gmail.com (J.L.)

[2] Key Laboratory of Micro-Nano Materials for Energy Storage and Conversion of Henan Province, College of Advanced Materials and Energy, Institute of Surface Micro and Nano Materials, Xuchang University, Xuchang 461000, China; tingtingli101@xcu.edu.cn

[3] Tianjin Key Laboratory of Food Science and Health, School of Medicine, Nankai University, Tianjin 300071, China

* Correspondence: xjdu@tust.edu.cn (X.D.); wangshuo@nankai.edu.cn (S.W.)

Experimental Section

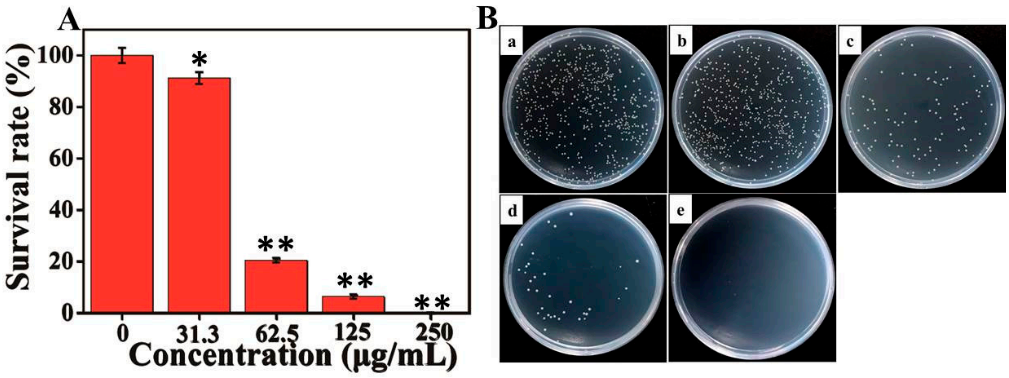

Figure S1. In vitro antibacterial activity test. (A) Cell survival rate of *Staphylococcus aureus* cells treated or untreated with MnO_2 nanosheets (31.5–250 μg/mL). (B) Photographs of the agar plates of *Staphylococcus aureus* treated or untreated with MnO_2 nanosheets, with a–e corresponding to the concentrations of MnO_2 nanosheets in Figure S1A (a–e: 0, 31.3, 62.5, 125, 250 μg/mL).
